# Notes and News

**Published:** 1894-09-01

**Authors:** 


### Notes and News.

Collected and Communicated.

A new ward, "the Clarence" ward, has been opened at the Alexandra Hospital, Woodhall Spa. Mr. E. Turner, High Sheriff of Lincolnshire, declared the ward open before a distinguished company. The institution is a national one, patients being received from all parts of the country. The Princess of Wales, who is a patroness of the institution, materially assisted in securing the necessary funds for the addition, by initiating a concert given by the Countess of Radnor for the purpose. £200 of the £600 expended was raised in this manner.

Sanitary inspectors are doubtlessly educated in the soundest principles of hygiene, and as a rule it is to be supposed that they start their career with the highest ideals of sanitation before them. Many of them early discover that knowledge is one thing, how to enforce its practice on others is another matter. The average standard of the locality in which they find themselves seems in some instances to be the test applied. This would certainly seem to be the case at Bermondsey, where a journeyman baker recently fell a victim to the conditions under which he worked. The baker fell dead at his work. An inquest was held. The chief sanitary inspector was present to give evidence, and his evidence revealed the fact that though the bakehouse in which the deceased worked was unfit for two men to occupy, that it was superior to some in the locality. The exposures as to the conditions of bakehouses and the employment of labour therein has not borne fruit enough yet to prevent this unfortunate man falling a victim at the age of twenty-eight to the severe conditions of his calling. Work frequently lasting twenty-one hours, passed in the heated atmosphere of a bakehouse but eight feet in height with scanty ventilation, and no holiday on Sunday, for a wage of twenty-seven shillings a-week is practically slavery. These workers who fall at their post might well rise up and demand a reasonable chance of life and health, and their just complaints should silence the clamouring cries for more wages and less work from a far more idle class, who are so needlessly backed by the sentimental amongst us and by the professional agitator.

The story which has appeared recently in the press of the candidate for employment in the Post Office who was deprived of fourteen teeth as a preliminary ceremony, is going the round of the foreign papers and causing some astonishment. It is probably the last misadventure of the kind which will befall candidates in future.

We are informed for the convenience of travellers to Buda-Pest and elsewhere on the Continent that a special arrangement has come into force between the South-Eastern Railway Company and the International Sleeping Car and European Express Trains Company by which a new special inquiry and ticket office has been opened at the entry to the Continental departure platform at Charing Cross Station. All Continental tickets, ordinary, train de luxe, and sleeping car, are issued at this office, which is in direct communication by private telephone with the latter company's chief office in Cockspur Street. Tickets for the Orient, Ostend-Vienna, Sud, Peninsula, Mediterranean, and Pyrennees expresses, as well as for the daily services to Bâle, Cologne, Lausanne, Rome, &c., are issued, when the cars are not already full, up to within five minutes of the departure of the corresponding trains from Charing Cross, or they may be reserved in advance. The early booking of berths for these services is always desirable, but for those passengers who may be unable to fix beforehand the date of their journey this arrangement will be of great advantage.

The plague at Hong Kong is now practically conquered, and this is due to the energy and self-denial of the British population, civil and military. Most interesting accounts are now forthcoming both as to the nature of the scourge, its treatment, and the attitude of the Chinese in the face of an epidemic of the kind. The nature of the disease appears to be much the same as that which was known as the Black Death, its characteristics only being modified by surroundings and climate. It appears that for very many years certain districts of China have been slightly infected, deaths occurring annually from it, whilst this year the disease mustered its forces in Canton, and was carried thence to Hong Kong. Canton is reported to have numbered 100,000 victims, and Hong Kong about 3,000, yet frightful as the condition of Canton was, numbers of the Chinese from Hong Kong fled thither rather than submit to the English sanitary measures, which they feared more than the plague itself. From the exodus and the plague, and the expenses connected with it, Hong Kong is practically ruined for the time being. This is the outcome of an ignorance and superstition which, a well-known English resident at Hong Kong points out, can best be overcome by the dissemination of medical knowledge through native medical men. There is a school of medicine in Hong Kong for Chinese students, but this cannot do the work it is quite capable of doing, and for which there are numbers of learners ready, for want of funds. For the lack of £5,000 a most valuable means is ineffectual of preventing a recurrence of the recent panic and ignorant behaviour of the population.

